# S100A4 Orchestrates Fibroblast Fate to Drive Fibrotic Remodeling

**DOI:** 10.1096/fj.202600886R

**Published:** 2026-06-13

**Authors:** Spring Li, Banruo Cai, Xu Zhang, Guoqiang Cai, Yong Zhou, Brant M. Wagener, Qiang Ding

**Affiliations:** ^1^ Division of Molecular and Translational Biomedicine, Department of Anesthesiology & Perioperative Medicine, Heersink School of Medicine University of Alabama at Birmingham Birmingham Alabama USA; ^2^ Section of Pulmonary Diseases, Critical Care and Environmental Medicine, School of Medicine Tulane University New Orleans Louisiana USA; ^3^ Birmingham Veterans Affairs Health Care System Birmingham Alabama USA

**Keywords:** ERK signaling, extracellular matrix remodeling, fibroblast activation, fibroblast fate, fibroblast survival, idiopathic pulmonary fibrosis, pulmonary fibrosis, S100A4

## Abstract

Fibroblast survival and dysregulated activation drive fibrotic diseases, including idiopathic pulmonary fibrosis (IPF). During physiological wound repair, fibroblasts are transiently activated to restore tissue integrity and are subsequently cleared by programmed cell death. In fibrotic disease, however, fibroblasts evade apoptosis and persist in a pathologically activated state. Although S100A4 has been implicated in fibrotic lung disease, the mechanisms by which S100A4 governs fibroblast fate and sustains profibrotic behavior remain unclear. Here, we identify S100A4 as a key regulator of apoptosis‐resistant, profibrotically activated fibroblasts through engagement of extracellular signal‐regulated kinase (ERK) signaling. In primary murine lung fibroblasts, S100A4 activates ERK, resulting in a coordinated program of fibroblast activation, including increased migration, extracellular matrix (ECM) contractility, stress fiber formation, and alpha‐smooth muscle actin (α‐SMA) induction. Functionally, S100A4 confers resistance to apoptosis induced by pro‐apoptotic and oxidative stress stimuli, as evidenced by reduced cleaved caspase‐3 and preserved cell viability. Pharmacological blockade of ERK signaling attenuates these responses, supporting ERK as an important downstream mediator of S100A4‐driven fibroblast activation and survival programs. Extending these findings to disease‐relevant contexts, bleomycin (BLM)‐induced lung injury in mice induces robust fibrotic remodeling, excessive collagen deposition, and transcriptional upregulation of S100A4. Consistently, primary lung fibroblasts from IPF patients exhibit elevated S100A4 expression, enhanced ERK activation, and increased α‐SMA expression, demonstrating conservation of this signaling axis across experimental models and human disease. Importantly, siRNA‐mediated knockdown of S100A4 in IPF fibroblasts suppresses ERK activation and attenuates expression of key profibrotic genes, indicating that S100A4 contributes to maintaining the fibrotic program in IPF fibroblasts. Collectively, these findings define a mechanistic link between S100A4‐mediated fibroblast survival and activation that drives pathological matrix remodeling and identify S100A4 and ERK as potential therapeutic targets in pulmonary fibrosis.

## Graphical abstract

Fibrotic stimuli induce S100A4 expression, promoting the activation of apoptosis‐resistant fibroblasts through engagement of ERK‐associated signaling. In fibroblasts, S100A4 orchestrates fibroblast fate by enhancing migration, promoting stress fiber formation, increasing ECM contractility, upregulating α‐SMA expression, and supporting survival, collectively sustaining persistent profibrotic remodeling. In experimental lung fibrosis and primary human IPF lung fibroblasts, S100A4 is upregulated together with elevated ERK activation and profibrotic gene expression. S100A4 inhibition or knockdown suppresses ERK activation and downstream profibrotic transcriptional responses, while ERK inhibition attenuates profibrotic behaviors and fibroblast survival, supporting a mechanistic role for S100A4‐mediated signaling in directing fibroblast fate and promoting progression toward pulmonary fibrosis.

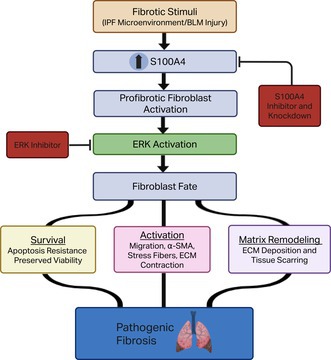

## 1 Introduction

Idiopathic pulmonary fibrosis (IPF) is the most common and lethal interstitial lung disease (ILD) [1], characterized by progressive, irreversible lung scarring and persistent alveolar injury [2, 3]. Activated fibroblasts, termed “myofibroblasts,” are the major effector cells responsible for excessive production of extracellular matrix (ECM) in IPF [4, 5]. Excessive ECM deposition distorts normal lung architecture, increases tissue stiffness, and impairs ventilation and gas exchange, ultimately leading to respiratory failure and death within 3–5 years [6]. Globally, the incidence of IPF is rising [7], posing substantial morbidity, mortality, and economic burden [8]. Diagnosis of IPF is challenging and often delayed, with symptom onset typically occurring between the ages of 50 and 70 years [9, 10]. While the exact cause of IPF remains unknown, advancing age, genetic predisposition, environmental exposures, and cigarette smoking are recognized risk factors that increase disease susceptibility [11].

During normal physiological conditions, fibroblasts undergo transient activation followed by apoptosis [12, 13]. In contrast, a defining pathogenic feature of IPF is the failure of fibroblasts to undergo apoptosis, resulting in their accumulation and differentiation into myofibroblasts. This establishes a self‐perpetuating profibrotic loop, characterized by continuous ECM production, expansion of fibrotic foci, and progressive tissue remodeling [14, 15, 16, 17, 18]. Dysregulated fibroblast turnover is therefore a central pathogenic mechanism and a critical point for therapeutic intervention.

S100A4 (metastasin‐1 and fibroblast‐specific protein 1), a calcium‐binding protein, is a member of the S100 family of proteins [19]. Although it lacks enzymatic activity, S100A4 functions as a molecular scaffold, regulating signaling networks via protein–protein interactions [20]. It has been implicated in cell survival, metastasis, inflammatory signaling, and calcium‐dependent responses [21, 22], and is upregulated in fibrotic lungs. Despite these associations, how S100A4 orchestrates fibroblast survival and fibroblast activation in pulmonary fibrosis remains incompletely understood.

In IPF, fibroblasts, which are the principal effector cells in IPF, differentiate into myofibroblasts and drive ECM deposition [23, 24, 25]. While S100A4 modulates cellular processes in diverse cell populations, including epithelial, neuronal, and cancer cells [26, 27], its direct role in fibroblast fate and the signaling pathways involved remains unresolved. Defining these mechanisms is critical, as sustained fibroblast activation and resistance to apoptosis are central drivers of IPF progression.

In this study, we evaluated whether S100A4 governs fibroblast survival and profibrotic activation via ERK signaling, a central pathway known to regulate proliferation, migration, differentiation, and apoptosis [28]. Using primary murine lung fibroblasts, we show that S100A4 activates ERK to enhance migration, stress fiber formation, ECM contraction, α‐SMA induction, and resistance to apoptosis. In vivo, BLM‐induced lung injury upregulates S100A4 expression during fibrotic remodeling. Importantly, primary fibroblasts from IPF patients recapitulate elevated S100A4 expression, ERK activation, and α‐SMA accumulation, demonstrating the translational relevance of this pathway. Consistently, siRNA‐mediated knockdown of S100A4 in IPF fibroblasts reduces ERK activation and attenuates expression of profibrotic genes, including ACTA2 and COL1A1, linking S100A4 to maintenance of the activated fibroblast state.

Collectively, these findings identify S100A4 as an important regulator of fibroblast survival and activation programs and highlight S100A4 and ERK signaling as promising therapeutic targets in IPF.

## 2 Materials and Methods

### 2.1 Animal Model of Lung Fibrosis

All animal procedures were approved by the local Institutional Animal Care and Use Committee. Lung fibrosis was induced in C57BL/6 mice (8–11 weeks) by intratracheal instillation of bleomycin (2 U/kg body weight in saline) under anesthesia, as described previously [29]. Control mice received saline alone. Lungs were harvested 21 days post‐instillation for histological and biochemical analyses. For histology, lungs were perfused with cold phosphate‐buffered saline, inflated with 10% neutral buffered formalin, fixed overnight, and embedded in paraffin [29, 30].

### 2.2 Antibodies and Other Reagents

The following antibodies were used: cleaved caspase‐3 (Cell Signaling Technology, Danvers, MA); phospho‐ERK (Santa Cruz Biotechnology, Santa Cruz, CA); ERK (Cell Signaling Technology); Cy3‐conjugated anti‐*α*‐SMA antibody (clone 1A4; Sigma‐Aldrich, Saint Louis, MO); α‐smooth muscle actin (SMA; American Research Products, Belmont, MA); S100A4 (Cell Signaling Technology); and glyceraldehyde 3‐phosphate dehydrogenase (GAPDH) (Cell Signaling Technology). Recombinant S100A4 was purchased from R&D Systems (Minneapolis, MN, USA). Chemicals were purchased from Sigma‐Aldrich (St. Louis, MO) and Fisher Scientific (Waltham, MA). Selective inhibitors, niclosamide and U0126, were purchased from Fisher Scientific, prepared as stock solutions in DMSO, and stored according to manufacturer instructions. Anti‐rabbit and anti‐mouse IgG peroxidase‐conjugated secondary antibodies were from Cell Signaling Technology.

### 2.3 Cells and Cell Culture

Primary murine lung fibroblasts were derived from 7‐ to 10‐week‐old C57BL/6 mice as described previously [31, 32, 33]. Briefly, lungs were aseptically removed, mechanically minced, and enzymatically digested with collagenase and DNase. Fibroblasts were isolated by differential adherence to tissue culture plastic and maintained in Dulbecco's modified Eagle's medium (DMEM) supplemented with 10% fetal bovine serum (FBS), 4 mM L‐glutamine, sodium pyruvate, and 100 units/mL penicillin/streptomycin. Experiments were performed using early‐passage fibroblasts (passages 2–9).

Normal human lung fibroblasts (NHLFs) were obtained from de‐identified lungs declined for transplantation, and primary idiopathic pulmonary fibrosis (IPF) patient fibroblasts were isolated from explanted lungs of IPF patients at the University of Alabama at Birmingham, as described previously [31]. Informed consent was obtained from all subjects, and protocols were approved by the Institutional Review Board (IRB). Human lung fibroblasts were maintained in MEM supplemented with 10% FBS, 4 mM L‐glutamine, sodium pyruvate, and 100 units/mL penicillin/streptomycin. Experiments were performed using early‐passage fibroblasts (passages 3–10).

### 2.4 Cell Migration Assay

Scratch‐wound (monolayer) migration assays were performed as described previously [31]. Primary murine lung fibroblasts were plated in serum‐free DMEM containing 1% BSA for 24 h. Cells were pretreated with 10 μg/mL mitomycin C to inhibit proliferation prior to wounding. A uniform scratch was made across the monolayer, and images were acquired immediately (0 h) and at the endpoint (24 h). Wound closure was quantified using ImageJ by measuring the reduction in wound area relative to the initial scratch width and normalized to untreated control fibroblasts. All experiments were conducted in three independent biological replicates, each performed in technical duplicate.

### 2.5 Cell Viability Assay

Cell viability was assessed using a Live/Dead Viability Kit (Thermo Fisher Scientific, Waltham, MA, USA) according to the manufacturer's instructions. Primary murine lung fibroblasts were seeded at 1 × 10^4^ cells per well in 6‐well plates and serum‐starved in DMEM supplemented with 1% BSA for 24 h. Cells were then treated with S100A4 (0.1 or 1 μg/mL), ± staurosporine (STS, 0.5 μM), and/or pretreated with the ERK inhibitor U0126 (10 μM, 30 min) as indicated. Following treatment, cells were washed with PBS and incubated with calcein AM (2 μM) and ethidium homodimer‐1 (4 μM) in PBS for 30 min at room temperature. Fluorescent images were acquired using an inverted fluorescence microscope (100× magnification). Images were analyzed in ImageJ to quantify live (calcein AM‐positive) and dead (EthD‐1‐positive) cells. The percentage of apoptotic cells was calculated as the ratio of dead cells to total cells. Experiments were performed in three independent biological replicates, each in technical duplicate.

### 2.6 Collagen‐Gel Contraction Assay

Collagen‐gel contraction assays were performed similarly as described previously [29, 34]. Briefly, 12‐well plates were coated with type I collagen gels prepared from rat tail collagen (BD Sciences, Bedford, MA) mixed with DMEM. Primary murine lung fibroblasts were embedded at 5 × 10^5^ cells per well. Gels were allowed to polymerize at room temperature for 30 min, then floated in serum‐free DMEM containing 1% BSA followed by ± niclosamide (10 μM, 30‐min pretreatment), followed by S100A4 (0.1 or 1 μg/mL). Cultures were incubated at 37°C, 5% CO_2_, for 24 h. Fibroblast‐mediated gel contraction was assessed by taking standardized photographs, and the gel surface area was quantified using ImageJ. Gel contraction was expressed as the percentage of remaining gel area relative to the initial gel area. All experiments were conducted in three independent biological replicates, each performed in technical duplicate.

### 2.7 Immunofluorescence Analysis

Immunofluorescence analysis was performed as previously described [32]. Primary murine lung fibroblasts were cultured on glass coverslips, fixed in 4% paraformaldehyde, and permeabilized in 1% Triton X‐100 in PBS at room temperature. Cells were stained with Cy3‐conjugated anti–*α*‐SMA monoclonal antibody (clone 1A4; Sigma‐Aldrich) to visualize cytoplasmic *α*‐SMA‐containing filaments, and nuclei were counterstained with Hoechst. Digital images were acquired using a fluorescence microscope at 200× magnification. For each coverslip, five randomly selected, non‐overlapping fields were analyzed in a blinded manner. The percentage of *α*‐SMA–positive cells relative to total Hoechst‐positive nuclei was quantified. Data represent three independent biological experiments, each performed in technical duplicate.

### 2.8 Immunoblot Analysis

Immunoblot analyses were performed as previously described [33]. Cells were lysed in RIPA buffer supplemented with protease and phosphatase inhibitors (10 μg/mL aprotinin, 10 μg/mL leupeptin, 400 μM phenylmethanesulfonyl fluoride (PMSF), and 400 μM sodium vanadate). Protein concentration of the whole‐cell lysate was determined by using a BCA kit (Pierce, Rockford, IL). Equal micrograms of whole‐cell lysates were electrophoresed on SDS‐PAGE, transferred to an Immobilon‐P PVDF membrane (Millipore), and probed with indicated primary and secondary antibodies. Membranes were developed using chemiluminescence and imaged with the ChemiDoc MP Imaging System (Bio‐Rad) and GeneSys imaging system. Band intensities were quantified using ImageJ, and expression levels were normalized to glyceraldehyde 3‐phosphate dehydrogenase (GAPDH) or total ERK, as appropriate. Data represent three independent biological experiments.

### 2.9 Analysis of Lung Fibrosis

The severity of lung fibrosis in bleomycin‐challenged mice was determined by lung collagen accumulation and morphometric fibrotic area quantification. Lung fibrotic areas were measured on H&E‐stained sections by morphometric methodologies (5 μm sections, paraffin‐embedded tissues). Lesional density was calculated as the percentage of lesional volume/total lung volume. Collagen deposition (5 μm lung tissue sections, paraffin‐embedded tissues) was localized by Masson's trichrome staining using a commercially available staining kit, according to the manufacturer's instructions (Poly Scientific, Bay Shore, NY).

### 2.10 Oxidative Stress‐Induced Apoptosis

Oxidative stress–induced apoptosis was assessed in primary murine lung fibroblasts. Cells were serum‐starved in DMEM supplemented with 1% BSA for 24 h prior to treatment. Cells were then pretreated with recombinant S100A4 (1 μg/mL). Following pretreatment, cells were exposed to hydrogen peroxide (H_2_O_2_, 200 μM) for up to 6 h. At the indicated time point, cells were lysed, and protein extracts were analyzed by immunoblotting. Apoptotic signaling was assessed by the detection of cleaved caspase‐3.

### 2.11 Real‐Time Quantitative PCR

Total RNA was extracted from lung fibroblasts or lung tissues using the RNeasy Mini Kit (Qiagen, Valencia, CA) according to the manufacturer's instructions. RNA integrity was confirmed using a NanoDrop spectrophotometer. The following primers were used: mouse S100A4, 5′‐TTGTGTCCACCTTCCACAAA‐3′ (sense) and 5′‐GCTGTCCAAGTTGCTCATCA‐3′ (antisense); human S100A4, 5′‐GATGAGCAACTTGGACAGCA‐3′ (sense) and 5′‐ACTCTTGGAAGTCCACCTCGT‐3′ (antisense); human ACTA2, 5′‐TCCGGGACATCAAGGAGAAACT‐3′ (sense) and 5′‐CCCATCAGGCAACTCGTAACTCT‐3′ (antisense); human COL1A1, 5′‐GAGGGCCAAGACGAAGACATC‐3′ (sense) and 5′‐CAGATCACGTCATCGCACAAC‐3′ (antisense); mouse glyceraldehyde 3‐phosphate dehydrogenase (GAPDH), 5′‐AACTTTGGCATTGTGGAAGG3′ (sense) and 5′‐ACACATTGGGGGTAGGAACA‐3′ (antisense); and human GAPDH, 5′‐AACATCATCCCTGCCTCTACTGG‐3′ (sense) and 5′‐GTTTTTCTAGACGGCAGGTCAGG‐3′ (antisense). Total RNA (1 μg) was reverse transcribed to cDNA using the LunaScript RT SuperMix (New England Biolabs, Ipswich, MA) according to the manufacturer's instructions. Quantitative PCR was performed using Luna Universal qPCR Master Mix (New England Biolabs, Ipswich, MA) on the CFX Opus 96 Real‐Time PCR System (Bio‐Rad, Hercules, CA). Reactions were performed in technical triplicates for each biological replicate. Gene expression was normalized to GAPDH and calculated using the 2^−∆∆Ct^ method, expressed relative to untreated or control conditions.

### 2.12 siRNA Transfection

Small interfering RNA (siRNA)–mediated knockdown of S100A4 was performed using the Lipofectamine 3000 Transfection Reagent (Thermo Fisher Scientific, Waltham, MA, USA) according to the manufacturer's instructions. Normal human lung fibroblasts and primary IPF patient fibroblasts were seeded to achieve ~60%–70% confluency at the time of transfection. S100A4‐targeting siRNA or non‐targeting scrambled control siRNA (Thermo Fisher Scientific) was diluted in serum‐free Opti‐MEM Reduced Serum Medium and mixed with Lipofectamine 3000 to allow complex formation at room temperature. Transfection complexes were added directly to cells in growth medium and incubated under standard culture conditions. Cells were harvested 48 h post‐transfection for downstream analyses.

### 2.13 Statistical Analysis

Statistical analyses were performed using RStudio (version 4.2.1). Data are presented as mean ± standard error of the mean (SEM). Comparisons between two groups were conducted using a two‐tailed Student's *t*‐test, while multiple‐group comparisons were performed using a one‐way ANOVA with Tukey's post hoc test, as appropriate. All experiments were performed with at least three independent biological replicates, with technical replication specified per assay. Quantitative analyses were performed in a blinded manner whenever subjective assessment was required. Statistical significance is indicated in the figures or figure legends and defined as *p < 0.05, **p < 0.01, and ***p < 0.001.

## 3 Results

### 3.1 S100A4 Promotes Fibroblast Migration via ERK‐Dependent Signaling

Fibroblast migration is a key cellular response to tissue injury that, while essential for effective wound repair, can also drive pathological tissue remodeling and fibrotic progression in the lung [4, 29, 33]. Persistent and dysregulated migration promotes fibroblast accumulation at sites of injury, amplifying ECM deposition and remodeling. S100A4, a calcium‐binding protein previously implicated in tumor metastasis [35]; is a known regulator of cytoskeletal dynamics and motility across multiple cell types, including epithelial and neuronal cells [36, 37], providing a mechanistic rationale for its role in directing fibroblast positioning within the injured lung microenvironment.

**FIGURE 1:**
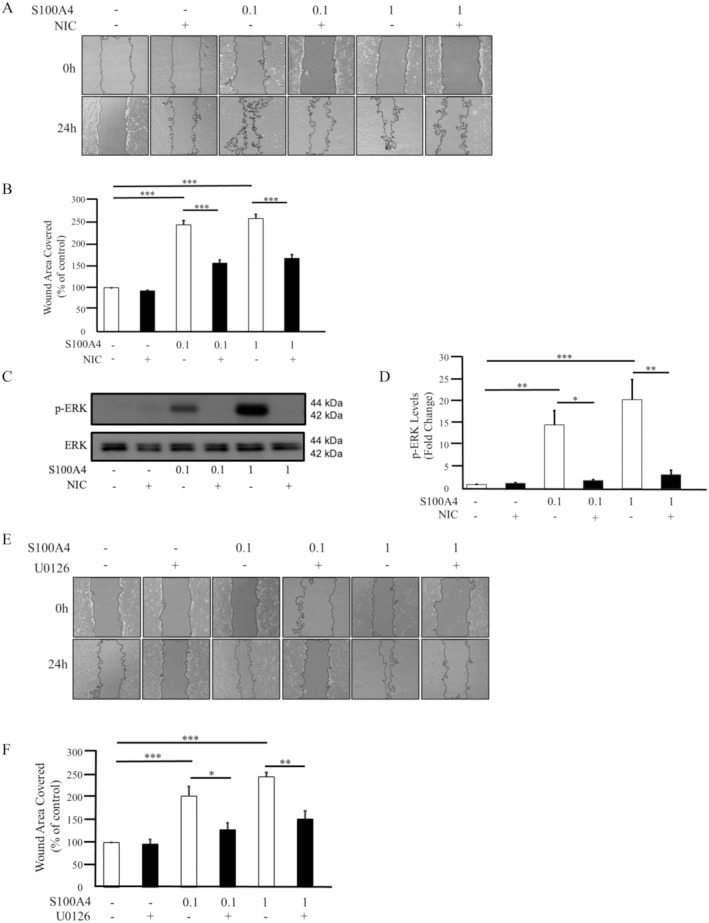
S100A4 promotes ERK‐dependent migration of primary murine lung fibroblasts. (A) Representative images from scratch‐wound assays in primary murine lung fibroblasts. Cells were serum‐starved for 24 h and pretreated with 10 μg/mL mitomycin C to inhibit proliferation, followed by S100A4 (0.1 or 1 μg/mL) ± niclosamide (NIC, 10 μM, 30min pretreatment). Wounds were imaged at 0 and 24 h. Images are representative of three independent biological experiments per condition (*n* = 3). (B) Quantification of wound closure from (A). Wound area is expressed as the percentage of area closed relative to untreated controls at 24 h. Bars represent the mean ± SEM of three independent biological experiments (*n* = 3). (C) Representative immunoblot analysis showing phospho‐ERK (p‐ERK) and total ERK expression in serum‐starved murine lung fibroblasts treated with S100A4 (0.1 or 1 μg/mL) ± niclosamide (10 μM, 30 min pretreatment) for 24 h. (D) Densitometric quantification of p‐ERK normalized to total ERK from (C). Bars represent the mean ± SEM of three independent biological experiments (*n* = 3). (E) Representative images of scratch‐wound assays in fibroblasts treated with S100A4 (0.1 or 1 μg/mL) ± the ERK inhibitor U0126 (10 μM, 30‐min pretreatment). Cells were serum‐starved as in (A). Images are representative of three independent biological experiments (*n* = 3). (F) Quantification of wound closure from (E). Data represent the mean ± SEM of three independent biological experiments (*n* = 3). Wound area is expressed as a percentage of the area closed at 24 h relative to untreated controls. In (A)–(F), data are expressed as mean ± SEM. Statistical significance was determined by one‐way ANOVA with Tukey's post hoc test. *p < 0.05, **p < 0.01, ***p < 0.001.

Serum‐starved primary murine lung fibroblasts were subjected to scratch‐wound assays in the presence of mitomycin C (10 μg/mL) to inhibit proliferation, followed by treatment with S100A4 (0.1 or 1 μg/mL) for 24 h (Figure 1A). Quantitative analysis revealed that S100A4 significantly accelerated wound closure (~243% and ~257% increases, respectively) relative to serum‐free controls (Figure 1B), demonstrating a robust, proliferation‐independent pro‐migratory response.

To determine whether this effect required active S100A4 signaling, fibroblasts were treated with S100A4 in the presence or absence of niclosamide (NIC), an FDA‐approved anthelmintic previously reported to disrupt S100A4‐associated signaling pathways in cancer models [38, 39, 40]. Niclosamide effectively attenuated S100A4‐induced migration (Figure 1B), indicating that S100A4‐mediated signaling is required to coordinate fibroblast motility.

Given the established role of ERK signaling in regulating cytoskeletal remodeling, migration, and cell survival [28], we next examined ERK activation. S100A4 treatment induced robust ERK phosphorylation in serum‐starved fibroblasts relative to untreated controls (Figure 1C,D). Importantly, niclosamide cotreatment significantly suppressed ERK phosphorylation, demonstrating that S100A4‐induced ERK activation is sensitive to pharmacological inhibition (Figure 1C,D).

Finally, to confirm ERK's role in S100A4‐driven fibroblast migration, fibroblasts were treated with U0126, an established MEK1/2 inhibitor that blocks ERK phosphorylation [41], in the presence or absence of S100A4, and wound closure was analyzed after 24 h (Figure 1E). ERK inhibition abrogated S100A4‐induced migration at both concentrations (Figure 1F), establishing ERK activation as a downstream effector of S100A4‐mediated fibroblast motility.

Collectively, these findings demonstrate that S100A4 regulates fibroblast migration in part through ERK signaling, contributing to positioning fibroblasts within injured tissue and supporting a profibrotic activation state.

### 3.2 S100A4 Promotes Fibroblast Survival Through ERK‐Dependent Anti‐Apoptotic Signaling

Fibroblast apoptosis is a critical regulatory checkpoint during normal wound resolution, limiting fibroblast accumulation following repair. In IPF, however, fibroblasts acquire resistance to apoptosis, resulting in persistent survival, pathological ECM deposition, and impaired tissue regeneration [12].

To assess whether S100A4 contributes to fibroblast survival, serum‐starved primary murine lung fibroblasts were pretreated with S100A4 (0.1 or 1 μg/mL) for 24 h prior to exposure to staurosporine (STS; 0.5 μM, 2 h), a potent inducer of apoptosis [42].

**FIGURE 2:**
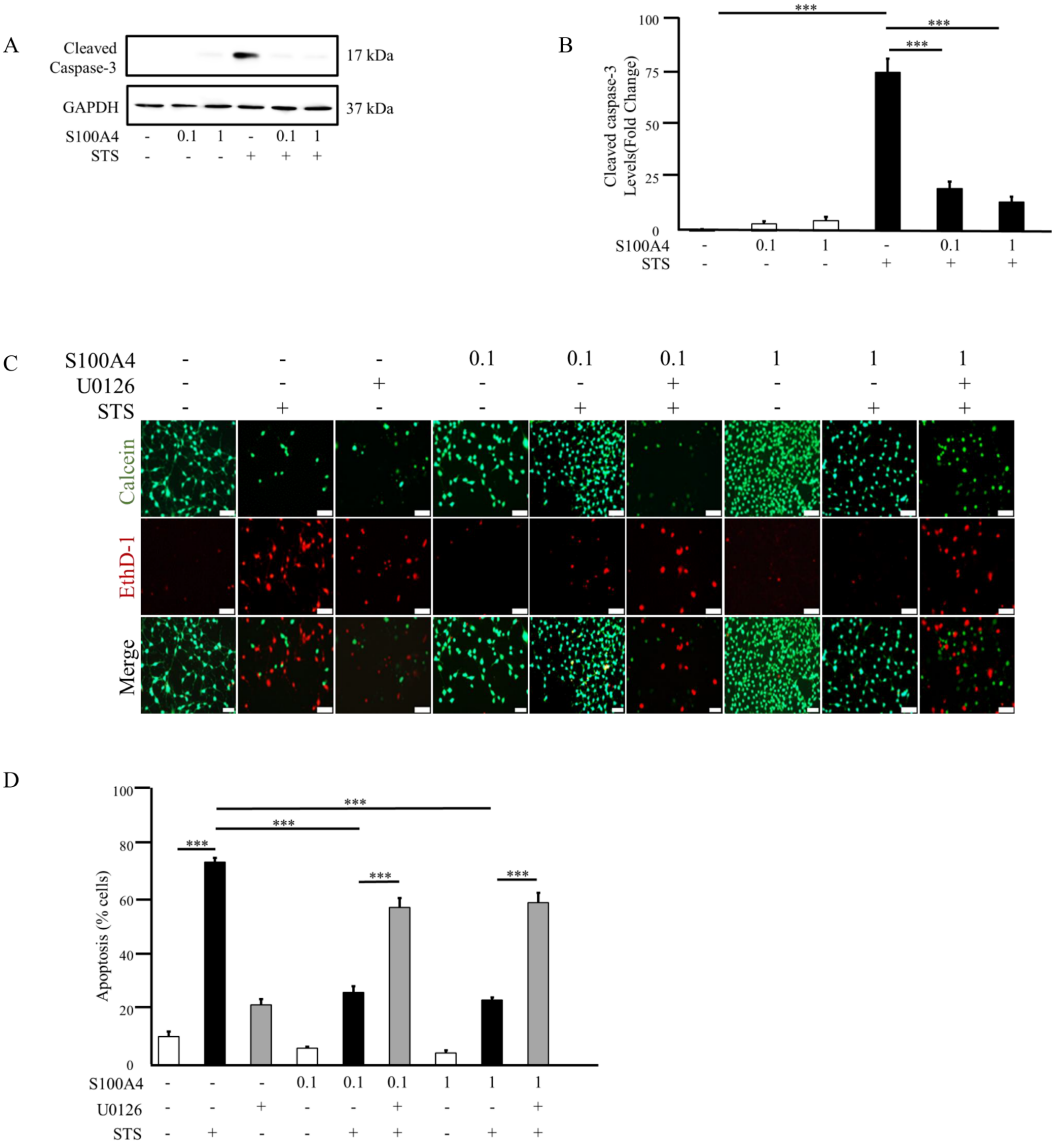
S100A4 enhances fibroblast survival by suppressing apoptosis through ERK signaling. (A) Representative immunoblot showing cleaved caspase‐3 expression in serum‐starved murine lung fibroblasts treated with S100A4 (0.1 or 1 μg/mL) ± staurosporine (STS, 0.5 μM). The antibody specifically recognizes the cleaved (active) form of caspase‐3. (B) Densitometric quantification of cleaved Caspase‐3 normalized to GAPDH. Bars represent the mean ± SEM of three independent biological experiments (*n* = 3). (C) Representative images of live/dead viability assays in serum‐starved murine lung fibroblasts treated with S100A4 (0.1 or 1 μg/mL) ± STS (0.5 μM). All cells were plated at comparable confluency to control cells prior to treatment and imaged 30 min post‐treatment. Cells were stained with 2 μM of calcein AM and 4 μM of ethidium homodimer‐1 (EthD‐1) mixture at room temperature. Calcein (green) labels live cells, whereas EthD‐1 (red) labels dead cells. Representative fields at 100× magnification. Scale bar: 20 μm. Images are representative of three independent experiments per condition (*n* = 3). (D) Quantification of the percentage of live and dead cells from (C). Five randomly selected, non‐overlapping fields per coverslip were analyzed in a blinded manner. Bars represent the mean ± SEM of three independent biological experiments (*n* = 3). In (A)–(D), data are expressed as mean ± SEM. Statistical significance was determined by one‐way ANOVA with Tukey's post hoc test. ***p < 0.001.

Apoptotic signaling was assessed biochemically by immunoblotting for cleaved caspase‐3, a canonical effector of apoptosis, and functionally by calcein AM/ethidium homodimer‐1 staining, which distinguishes live (green) versus dead (red) cells, enabling quantitative assessment of fibroblast viability under apoptotic stress (Figure 2).

STS treatment significantly increased cleaved caspase‐3 levels relative to vehicle controls (Figure 2A,B). Pretreatment with S100A4 significantly attenuated STS‐induced caspase‐3 cleavage in a concentration‐dependent manner (Figure 2A,B), demonstrating a robust anti‐apoptotic effect. Similarly, calcein AM/ethidium homodimer‐1 assays confirmed functional protection, with STS inducing ~75% cell death, whereas S100A4 reduced STS‐induced apoptosis to ~30% (0.1 μg/mL) and ~22% (1 μg/mL) (Figure 2C,D), indicating substantial preservation of fibroblast viability under apoptotic stress.

We expanded our experimental approach to include an oxidative stress model using hydrogen peroxide (H_2_O_2_) [43]. S100A4 significantly attenuated H_2_O_2_‐induced caspase‐3 activation (Figure S1A,B), consistent with its anti‐apoptotic effects observed under STS challenge. Our data suggest that S100A4 attenuates apoptosis across distinct apoptotic stress modalities, including under both STS and H_2_O_2_ conditions.

Given our observation that S100A4 activates ERK signaling (Figure 1C), we next evaluated whether this survival effect was ERK‐dependent. Pharmacological inhibition of ERK with U0126 attenuated S100A4‐mediated protection, restoring apoptotic cell death to levels comparable to STS alone (Figure 2C,D). These findings support ERK signaling as a downstream mediator of S100A4‐associated fibroblast survival.

Together, these findings indicate that S100A4 promotes an apoptosis‐resistant fibroblast phenotype in part through ERK activation, which may contribute to fibroblast persistence at sites of injury and support fibrotic remodeling.

### 3.3 S100A4 Drives Myofibroblast Differentiation via α‐SMA Induction and Cytoskeletal Remodeling

During both normal wound healing and the development of fibrosis, fibroblasts undergo a phenotypic transition into activated fibroblasts, termed myofibroblasts. These cells serve as the primary effectors of ECM production and tissue remodeling [17, 18, 29, 44, 45]. This phenotypic transition is characterized by increased cell size, enhanced contractility, cytoskeletal reorganization, and the formation of prominent actin stress fibers [46]. Alpha‐smooth muscle actin (α‐SMA) is a widely recognized phenotypic marker of myofibroblast differentiation, and its incorporation into stress fibers reflects enhanced contractile capacity and sustained fibroblast activation [44].

**FIGURE 3:**
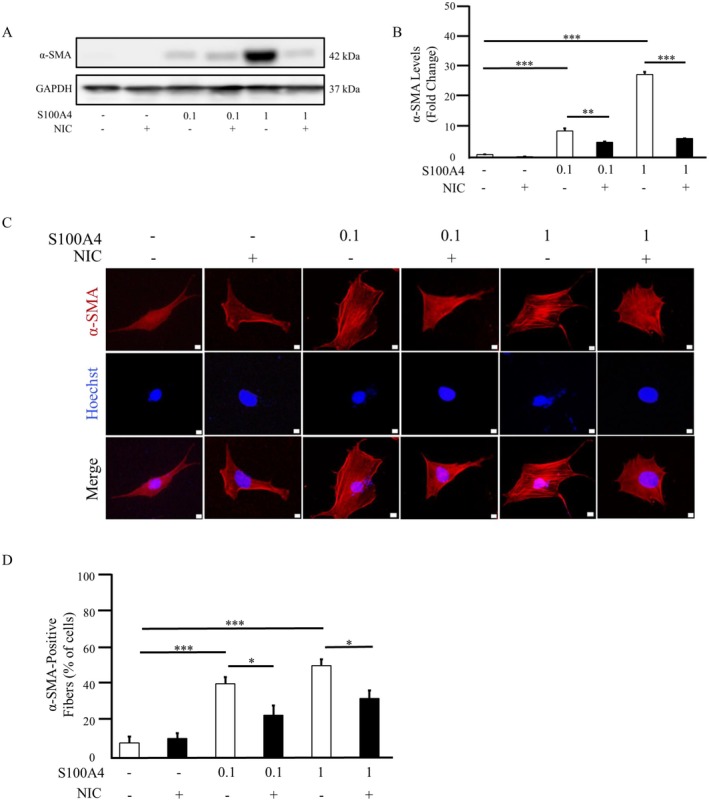
S100A4 drives myofibroblast differentiation of primary murine lung fibroblasts. (A) Representative immunoblot showing α‐SMA expression in serum‐starved murine lung fibroblasts treated with S100A4 (0.1 or 1 μg/mL) ± niclosamide (10 μM, 30 min pretreatment) for 24 h. (B) Densitometric quantification of α‐SMA normalized to GAPDH from (A). Bars represent the mean ± SEM of three independent biological experiments (*n* = 3). (C) Representative immunofluorescent images of primary murine lung fibroblasts plated on coverslips and treated with ± niclosamide (10 μM, 30 min pretreatment), followed by S100A4 (0.1 or 1 μg/mL). Cells were fixed and immunostained with Cy‐3‐labeled monoclonal antibody toward α‐SMA (red) and Hoechst for nuclei (blue). Images were acquired at 200× magnification. Scale bar: 10 μm. Images are representative of three independent biological experiments per condition (*n* = 3). (D) Quantification of the percentage of cells displaying highly organized, thickened *α*‐SMA–containing fibers from (C). Five randomly selected, non‐overlapping fields per coverslip were analyzed in a blinded manner. Bars represent the mean ± SEM of three independent biological experiments (*n* = 3). In (A)–(D), data are expressed as mean ± SEM. Statistical significance was determined by one‐way ANOVA with Tukey's post hoc test. *p < 0.05, **p < 0.01, ***p < 0.001.

To determine whether S100A4 promotes fibroblast‐to‐myofibroblast differentiation and associated cytoskeletal remodeling, primary murine lung fibroblasts were treated with S100A4 (0.1 or 1 μg/mL). S100A4 significantly increased α‐SMA protein levels in a concentration‐dependent manner, ~9‐fold and ~27‐fold relative to controls (Figure 3A,B). Co‐treatment with niclosamide markedly attenuated S100A4‐induced α‐SMA upregulation, indicating that S100A4‐driven differentiation requires intact downstream signaling.

Next, to determine whether elevated *α*‐SMA expression translated into structural myofibroblast features, we quantified *α*‐SMA–positive stress fiber formation. S100A4 significantly increased the proportion of fibroblasts containing organized *α*‐SMA‐positive stress fibers (Figure 3C, panels 3 and 5), from ~8% in control fibroblasts to ~41% and ~53% following treatment with 0.1 and 1 μg/mL S100A4, respectively (Figure 3D). These findings demonstrate that S100A4 induces both molecular and cytoskeletal features characteristic of myofibroblast differentiation.

Consistent with its inhibitory effects on *α*‐SMA expression, niclosamide co‐treatment significantly suppressed S100A4‐induced stress fiber formation (Figure 3C, panels 4 and 6), decreasing the proportion of cells exhibiting *α*‐SMA–positive stress fibers to ~23% and ~33%, respectively (Figure 3D). This pharmacological inhibition further confirms that S100A4‐driven cytoskeletal remodeling and contractile activation are dependent on signaling pathways inhibited by niclosamide.

Together, these results identify S100A4 as a regulator of fibroblast fate, promoting differentiation into highly contractile myofibroblasts through induction of *α*‐SMA expression and cytoskeletal remodeling.

### 3.4 S100A4 Promotes Fibroblast‐Mediated ECM Contraction

Activated fibroblasts are highly contractile cells that pull on collagen fibers to facilitate wound closure [47]. However, sustained or dysregulated contractility is a defining functional hallmark of myofibroblast activation and contributes directly to pathological tissue remodeling, matrix stiffening, and progressive scar formation during fibrosis [48]. Persistent fibroblast‐mediated ECM contraction thus reinforces a self‐sustaining contractile, profibrotic niche.

**FIGURE 4:**
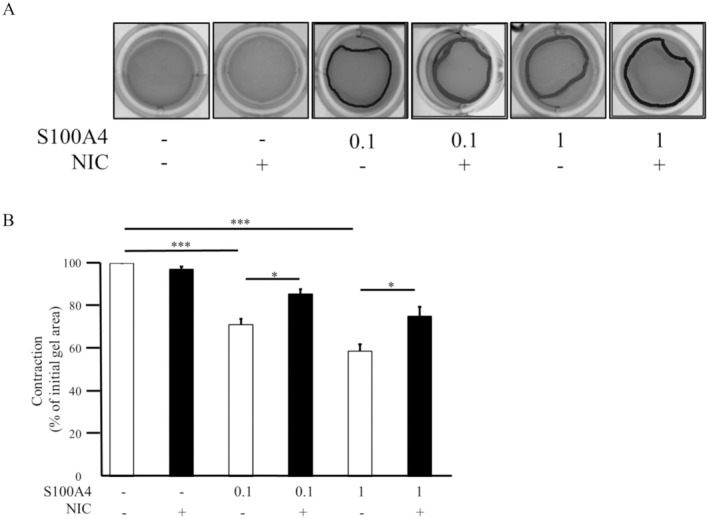
S100A4 enhances fibroblast‐driven ECM remodeling through collagen gel contraction. (A) Representative images of collagen‐gel contraction by primary murine lung fibroblasts. Cells were suspended in DMEM, mixed with collagen solution (rat tail collagen) (500 μL per well), allowed to polymerize at room temperature for 30 min, and then floated in serum‐free media (DMEM, 1% BSA) ± niclosamide (10 μM, 30‐min pretreatment) followed by S100A4 (0.1 or 1 μg/mL). Gels were incubated at 37°C, 5% CO_2_ for 24 h. Images are representative of three independent biological experiments per condition (*n* = 3). (B) Quantification of collagen‐gel contraction from (A). Contraction is expressed as the percentage of gel area relative to the initial gel area, with lower values indicating increased contractile activity. Bars represent the mean ± SEM of three independent biological experiments (*n* = 3). Statistical significance was determined by one‐way ANOVA with Tukey's post hoc test. *p < 0.05, ***p < 0.001.

To assess whether S100A4 regulates fibroblast‐mediated ECM remodeling, we performed three‐dimensional collagen gel contraction assays. S100A4 treatment significantly enhanced fibroblast‐mediated ECM contraction relative to controls (Figure 4A, panels 3 and 5 vs. panel 1). After 24 h, fibroblasts treated with 0.1 μg/mL S100A4 contracted by ~29%, while 1 μg/mL S100A4 induced a ~41% reduction in gel area (Figure 4B).

In contrast, inhibition of S100A4 activity with niclosamide markedly reduced fibroblast‐mediated ECM contraction (Figure 4A, panels 4 and 6). Niclosamide co‐treatment attenuated S100A4‐induced gel contraction at both concentrations (Figure 4B), demonstrating that S100A4 activity is required for sustained fibroblast contractile activity.

Collectively, these findings support S100A4 as a modulator of fibroblast‐mediated ECM remodeling, linking molecular activation to increased mechanical force generation that contributes to fibrotic matrix remodeling.

### 3.5 S100A4 Is Transcriptionally Upregulated In Vivo During Bleomycin‐Induced Lung Fibrosis

Having demonstrated that S100A4 enhances fibroblast migration, contractility, and differentiation in vitro, we examined whether S100A4 is induced in vivo during lung injury and fibrotic remodeling. Using a murine model of BLM‐induced pulmonary fibrosis, lungs were harvested 21 days after intratracheal BLM administration.

**FIGURE 5:**
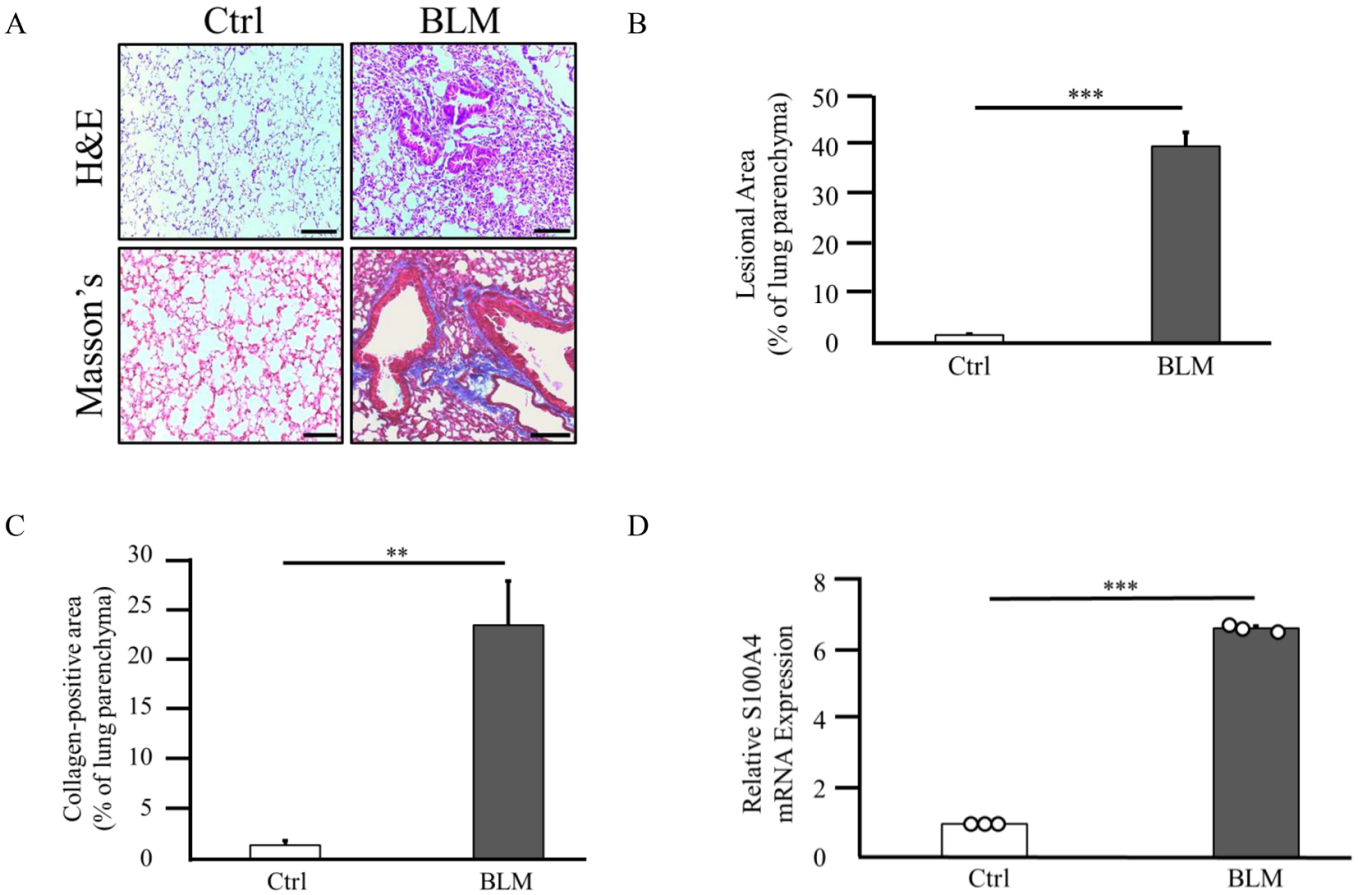
Bleomycin induces progressive lung fibrosis and collagen deposition associated with S100A4 upregulation. (A) Representative images of hematoxylin and eosin (H&E)‐stained (top) and Masson's trichrome stained (collagen deposition, blue; bottom) lung sections from control (Ctrl) and bleomycin‐challenged (BLM) mice harvested 21 days post‐intratracheal administration. Images were acquired at 100× magnification. Scale bar: 20 μm. Images are representative of 4–5 non‐overlapping fields per mouse from *n* = 3–5 mice per group. (B) Quantification of fibrotic lesional area (%) from H&E‐stained images. (C) Quantification of collagen‐positive area (%) from Masson trichrome‐stained images. (D) Relative S100A4 mRNA expression in lung tissue from Ctrl and BLM mice as determined by RT‐qPCR. Expression was normalized to GAPDH and expressed relative to Ctrl. In (A)–(D), data are presented as mean ± SEM. Statistical significance was determined using a two‐tailed unpaired Student's *t*‐test: **p < 0.01, ***p < 0.001.

Histological analysis of hematoxylin and eosin‐stained sections revealed extensive alveolar remodeling and parenchymal thickening, with pronounced loss of normal alveolar architecture, a hallmark of fibrotic remodeling (Figure 5A, top panel). Quantitative morphometric analysis of H&E‐stained sections confirmed substantial expansion of fibrotic regions, with BLM‐treated lungs occupying ~40% of total lung parenchyma relative to ~3% in saline controls (Figure 5B).

To assess ECM deposition, Masson's trichrome staining revealed robust collagen accumulation throughout the interstitium of BLM‐treated lungs, whereas saline‐treated controls exhibited minimal collagen deposition (Figure 5A, bottom panel). Quantification of collagen‐positive area revealed a significant increase in fibrotic burden, with collagen occupying ~23% of total lung parenchyma in BLM‐exposed mice compared with negligible levels in saline controls (Figure 5C).

Concomitantly, RT‐qPCR analysis of lung tissue revealed a significant ~7‐fold increase in S100A4 mRNA expression in BLM‐treated lungs relative to saline controls (*n* = 3, Figure 5D), providing direct in vivo evidence of S100A4 transcriptional induction during fibrotic lung remodeling.

Together, these findings establish a robust in vivo pathology characterized by architectural distortion, excessive collagen deposition, and concurrent upregulation of S100A4, supporting its role as an injury‐responsive regulator of fibroblast activation in pulmonary fibrosis.

### 3.6 Conserved S100A4‐ERK Signaling Underlies Fibroblast Activation in IPF Fibroblasts

**FIGURE 6:**
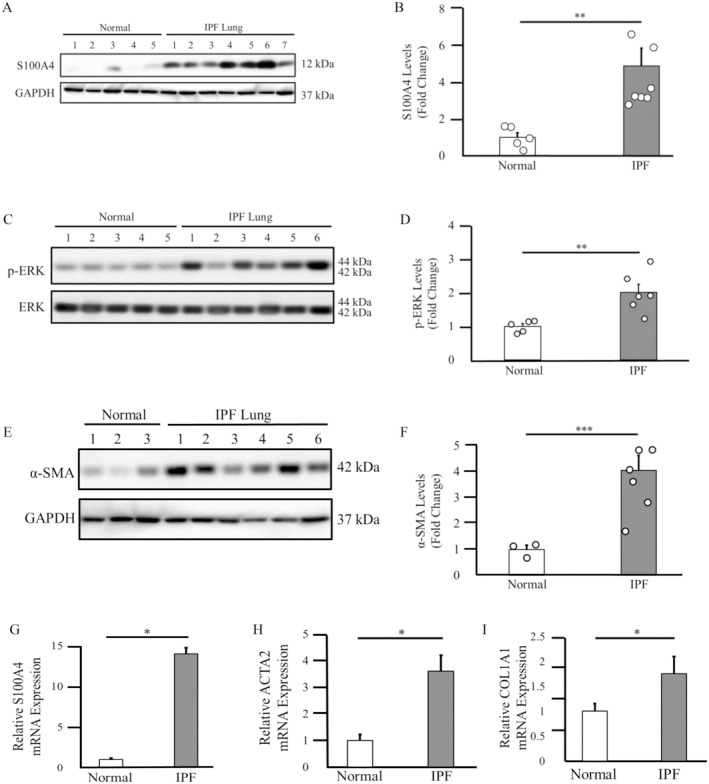
Conserved S100A4‐ERK signaling and myofibroblast activation in primary human IPF fibroblasts. (A) Representative immunoblots of S100A4 protein expression in primary lung fibroblasts from healthy donors (normal, *n* = 5) and IPF patients (*n* = 7). (B) Densitometric quantification of S100A4 normalized to GAPDH from (A). Bars represent mean ± SEM; *n* = number of independent donor samples. (C) Representative immunoblot analysis showing phospho‐ERK (p‐ERK) and total ERK expression in primary lung fibroblasts from healthy donors (normal, *n* = 5) and IPF patients (*n* = 6). (D) Densitometric quantification of p‐ERK normalized to total ERK from (C). Bars represent mean ± SEM; *n* = number of independent donor samples. (E) Representative immunoblot of α‐SMA in normal (*n* = 3) and IPF fibroblasts (*n* = 6). (F) Densitometric quantification of α‐SMA normalized to GAPDH from (E). Bars represent mean ± SEM; *n* = number of independent donor samples. RT‐qPCR analysis of (G) S100A4, (H) ACTA2, and (I) COL1A1 mRNA expression. Expression was normalized to GAPDH and presented relative to normal fibroblasts. In (A)–(I), data are presented as mean ± SEM. Statistical significance was determined using a two‐tailed unpaired Student's *t*‐test: *p < 0.05, **p < 0.01, ***p < 0.001.

To assess whether mechanisms observed in murine fibroblasts are conserved in human disease, we examined S100A4 expression and downstream signaling in primary lung fibroblasts derived from patients with IPF and healthy donors. Immunoblot analyses revealed a significant upregulation of S100A4 protein in IPF fibroblasts (*n* = 7) relative to normal controls (*n* = 5), with a mean increase of ~5‐fold (range ~3–7; Figure 6A,B), indicative of an activated fibroblast state.

Concordantly, phosphorylated ERK levels were significantly increased in IPF fibroblasts (*n* = 6) relative to controls (*n* = 5), with a mean increase of ~2‐fold (range ~1–3; Figure 6C,D), indicating coordinated activation of ERK signaling in S100A4‐enriched fibrotic fibroblasts.

To further determine whether S100A4 contributes to ERK activation in human IPF fibroblasts, S100A4 expression was silenced using siRNA. Knockdown of S100A4 in IPF fibroblasts significantly reduced ERK phosphorylation toward baseline levels relative to scrambled siRNA controls (Figure S2A,B), supporting a role for endogenous S100A4 in maintaining ERK activation in the fibrotic state.

Consistent with enhanced myofibroblast differentiation observed in murine systems, *α*‐SMA immunoblot analysis revealed a significant elevation in IPF samples (*n* = 6) relative to normal controls (*n* = 3), with a mean increase of ~4‐fold (range ~1–5; Figure 6E,F), indicating a myofibroblast‐differentiated phenotype in IPF fibroblasts.

To determine whether S100A4 upregulation occurs at the transcriptional level, RT‐qPCR was performed in primary lung fibroblasts. S100A4 mRNA expression was significantly elevated in IPF fibroblasts (~14‐fold increase; Figure 6G), corroborating protein‐level findings and demonstrating transcriptional induction.

In parallel, expression of key profibrotic and ECM‐associated genes, including ACTA2 and COL1A1, was significantly increased in IPF fibroblasts relative to healthy controls (~3.6‐fold and ~1.75‐fold, respectively; Figure 6H,I), consistent with activation of a coordinated fibrotic gene program.

Transfection with S100A4‐targeting siRNA (siS100A4) efficiently reduced S100A4 mRNA expression in IPF fibroblasts (~92% knockdown relative to scrambled siRNA controls; Figure S3A). Knockdown of S100A4 significantly reduced expression of profibrotic markers ACTA2 and COL1A1 (~46% and ~69% decrease, respectively; Figure S3B,C), indicating suppression of the fibrotic transcriptional program.

Collectively, these results support a conserved role for S100A4‐mediated signaling in fibroblast activation in IPF. The coordinated elevation of S100A4, ERK activation, and α‐SMA expression is consistent with a profibrotic activation state in IPF fibroblasts and links S100A4 with fibroblast activation and profibrotic gene expression in pulmonary fibrosis.

## 4 Discussion

Our study establishes S100A4 as an important orchestrator of fibroblast survival and profibrotic activation in primary murine lung fibroblasts. Mechanistically, S100A4 enhances fibroblast viability via the ERK signaling cascade, resulting in reduced apoptosis, as evidenced by decreased cleaved caspase‐3 levels and a lower proportion of apoptotic cells. Pharmacological inhibition of ERK signaling using U0126 restores apoptotic susceptibility, supporting ERK as a downstream mediator of S100A4‐associated survival signaling. Beyond survival, S100A4 drives multiple profibrotic functions, including migration, contractility, and differentiation into α‐SMA‐positive myofibroblasts. Niclosamide treatment significantly attenuates these phenotypes, demonstrating that S100A4's profibrotic activity is pharmacologically targetable.

Collectively, these findings position S100A4 as a dual‐function orchestrator of fibroblast activation and survival programs that drive pathological remodeling in the lung.

Persistent fibroblast and myofibroblast survival are conserved drivers of fibrosis across organs, including the kidney, lung, and liver [49, 50, 51]. S100A4 contributes to this apoptosis‐resistant, profibrotic phenotype, in part through ERK‐associated signaling, and is associated with functional fibrotic outcomes in both murine and human fibroblasts.

There is currently no cure for IPF; management focuses on slowing disease progression, preserving lung function, and alleviating symptoms. The two globally approved therapies, nintedanib and pirfenidone, demonstrate modest antifibrotic activity [52, 53]. Nintedanib, a tyrosine kinase inhibitor, targets growth factor pathways, fibroblast growth factor (FGF), platelet‐derived growth factor (PDGF), and vascular endothelial growth factor (VEGF) [54] and was originally developed for oncology‐based anti‐angiogenic applications. Clinical trials demonstrated reduced acute exacerbation rates, slower lung function decline, and lower mortality [55]. Pirfenidone also exhibits antifibrotic activity and anti‐inflammatory effects, reducing fibroblast proliferation and ECM synthesis through inhibition of growth factors and TGF‐β signaling [56, 57]. Although these drugs slow IPF progression [52, 53], modest efficacy and notable side effects [58, 59] highlight the urgent need for mechanistically novel therapies targeting fibroblast persistence and activation.

Given the limitations of current therapies, a detailed understanding of fibroblast survival and the mechanisms that maintain the profibrotic phenotype is critical. Identifying molecular regulators that couple survival signaling to sustained fibroblast activation represents a key unmet need in IPF. Potential approaches include promoting apoptosis of activated fibroblasts or reprogramming them toward a quiescent state. For example, in liver fibrosis, antibody‐targeted fibroblast and myofibroblast apoptosis reduced fibrosis [60], and in cardiac fibrosis, the excision of activated myofibroblasts during chronic stress conferred cardioprotection, while removal of stressors promoted reversion to a quiescent, less active state [61]. Our previous work also demonstrated that targeting Src kinases mitigates myofibroblast differentiation and ECM production, highlighting multiple avenues for suppressing fibroblast activation [32].

Extracellular signal‐regulated kinase (ERK), a central pathway belonging to the mitogen‐activated protein kinase (MAPK) family of serine/threonine protein kinases, translates extracellular stimuli into cellular responses, including migration, proliferation, differentiation, metabolism, and survival. Activation of ERK promotes cell survival and is important for fibroblast function [62, 63], and our data establish S100A4 as a modulator of ERK in both murine and human fibroblasts. Recent studies on cardiac fibrosis identified artesunate (ART) as a suppressor of TGF‐β‐induced fibroblast activation, acting in part through inhibition of ERK [64], suggesting a conserved role for ERK in fibroblast‐driven fibrosis across organ systems. Our findings support ERK as a mediator of profibrotic fibroblast responses in the lung, providing a framework for exploring ERK‐targeted interventions in diverse fibrotic contexts [64, 65, 66, 67].

S100A4 has been reported in other systems to function in both intracellular and extracellular contexts, with proposed autocrine and paracrine signaling roles in cancer settings [35, 37, 68, 69]. Several studies further suggest that extracellular S100A4 may engage cell‐surface receptors such as RAGE, which is a known upstream activator of MAPK signaling, including ERK, in multiple cell types. In addition, S100A4 has been implicated in other receptor‐mediated signaling pathways that converge on MAPK/ERK activation [70, 71, 72]. However, the precise upstream mechanisms linking S100A4 to ERK activation in lung fibroblasts were not directly investigated in the present study. Accordingly, the spatial and functional mode of S100A4 signaling in lung fibroblasts remains unresolved and represents an important area for future investigation.

Despite lacking intrinsic enzymatic activity, S100A4 functions as a pivotal modulator of fibrotic signaling via protein–protein interactions and ERK regulation [73]. By promoting myofibroblast differentiation, migration, contractility, and apoptosis resistance, S100A4 emerges as a compelling therapeutic target. Preclinical studies demonstrate that targeting S100A4 or ERK mitigates fibrosis [65, 74, 75, 76, 77], and S100A4‐neutralizing antibodies reduce dermal fibrosis, myofibroblast numbers, and collagen accumulation in systemic sclerosis models [74, 78], highlighting translational potential.

Importantly, we show that S100A4 signaling is conserved in human IPF fibroblasts, establishing translational relevance. Fibroblast heterogeneity in IPF is increasingly recognized, with distinct transcriptional and functional fibroblast subpopulations contributing to disease progression. Consistent with this concept, we observed variability in S100A4 and α‐SMA expression across primary human IPF fibroblast lines. Whether S100A4‐associated signaling is uniformly represented across all fibroblast subpopulations remains an important question for future investigation. This variability is consistent with enrichment of S100A4 signaling within specific pathogenic fibroblast states in the fibrotic lung microenvironment.

Together, our results define the S100A4‐mediated signaling associated with fibroblast survival and profibrotic activation, integrating molecular signaling with functional phenotypes and disease progression.

While ERK signaling represents a downstream mediator of S100A4‐driven fibroblast activation, additional signaling pathways may also contribute to the observed phenotypes. We acknowledge several limitations in our experimental approach. Niclosamide is a pleiotropic compound that has been used to assess S100A4‐associated biology [38, 40, 79], but it is not pathway‐specific and likely affects multiple signaling networks beyond ERK. Accordingly, phenotypes suppressed by niclosamide, including ECM remodeling, contractility, and myofibroblast differentiation, cannot be attributed exclusively to ERK signaling. Thus, niclosamide should be interpreted as a broad inhibitor of S100A4‐associated signaling responses rather than a selective mechanistic probe for ERK activity. This distinction is important in interpreting ERK contribution, as niclosamide‐sensitive effects likely reflect combined disruption of multiple S100A4‐associated pathways rather than ERK signaling alone, thereby limiting direct attribution of these phenotypes to ERK alone. ERK dependence was directly established using the selective MEK1/2 inhibitor U0126 in migration and apoptosis assays, whereas niclosamide was used to broadly suppress S100A4‐associated phenotypes rather than to specifically assess ERK signaling.

Interventional approaches targeting S100A4 in vivo would further strengthen the mechanistic and translational relevance of our findings in the bleomycin model. Addressing this limitation will be important for confirming the functional contribution of S100A4 to fibrotic progression within the intact lung microenvironment. Such studies, including fibroblast‐specific deletion of S100A4, would complement our pharmacological and in vitro findings by enabling direct evaluation of S100A4‐dependent mechanisms in vivo, thereby providing a more comprehensive understanding of its role in fibrotic remodeling.

Future studies should therefore focus on elucidating the broader S100A4 signaling network, including potential parallel pathways, and evaluating the in vivo relevance of this axis, as well as assessing the potential for combination therapies with current antifibrotics. Additional studies using single‐cell and lineage‐resolved approaches will be important to determine whether S100A4 expression is enriched within distinct profibrotic fibroblast states in the IPF microenvironment. Further work is also needed to define the signaling context and compartmentalization of S100A4 activity.

Future studies should also determine whether ERK activation alone is sufficient to recapitulate S100A4‐driven fibroblast phenotypes. This should be addressed using gain‐of‐function approaches, including constitutively active MEK/ERK systems, alongside complementary loss‐of‐function approaches, including dominant‐negative ERK strategies, to further define the contribution of ERK signaling to the observed profibrotic phenotypes.

Targeting the S100A4 and ERK may offer a next‐generation strategy for antifibrotic interventions to halt or reverse fibrosis across multiple organ systems and fibrotic diseases.

In summary, our study positions S100A4 as a dual‐function orchestrator of fibroblast survival and profibrotic activation involving ERK signaling, driving apoptosis resistance, myofibroblast differentiation, migration, and ECM remodeling. Conserved signaling in human IPF fibroblasts underscores translational relevance, establishing S100A4 and ERK as promising targets for next‐generation antifibrotic therapies.

## Author Contributions

S.L. and Q.D. conceptualized and designed the research; S.L. conducted the experiments, performed data analysis and interpretation, and prepared the figures with input from all authors; X.Z., G.C., and B.C. assisted with experimental work. S.L. wrote the original draft of the manuscript; S.L., Q.D., B.M.W., X.Z., B.C., Y.Z., and G.C. contributed to the writing and/or critical review of the manuscript and approved the final version. Q.D. supervised the study and provided financial support.

## Funding

This work was supported by HHS|NIH|National Heart, Lung, and Blood Institute (NHLBI) (R01HL085324), U.S. Department of Veterans Affairs (VA) (I01BX006354), and HHS|NIH|National Institute on Aging (NIA) (RF1AG094716).

## Conflicts of Interest

The authors declare no conflicts of interest.

## Supporting information


**Figure S1:** S100A4 attenuates hydrogen peroxide–induced apoptotic signaling in primary murine lung fibroblasts. Serum‐starved primary murine lung fibroblasts were pretreated with S100A4 (1 μg/mL) prior to exposure to hydrogen peroxide (H_2_O_2_, 200 μM). (A) Representative immunoblot showing cleaved caspase‐3 expression following H_2_O_2_‐induced oxidative stress ± S100A4 pretreatment. (B) Densitometric quantification of cleaved caspase‐3 normalized to GAPDH. Data are presented as mean ± SEM of three independent biological experiments (*n* = 3). Statistical significance was determined by One‐way ANOVA with Tukey's post hoc test. *p < 0.05 and **p < 0.01.
**Figure S2:** S100A4 knockdown attenuates ERK phosphorylation in primary human IPF fibroblasts. Primary human IPF fibroblasts were transfected with scrambled siRNA (scr siRNA) or S100A4‐targeting siRNA (siS100A4). (A) Representative immunoblot analysis showing phospho‐ERK (p‐ERK) and total ERK expression. (B) Densitometric quantification of p‐ERK normalized to total ERK from (A). Bars represent mean ± SEM. Statistical significance was determined by One‐way ANOVA with Tukey's post hoc test. *p < 0.05.
**Figure S3:** siRNA‐mediated S100A4 knockdown attenuates profibrotic gene expression in primary human IPF fibroblasts. RT‐qPCR analysis of (A) S100A4, (B) ACTA2, and (C) COL1A1 mRNA expression in IPF fibroblasts transfected with scrambled siRNA (scr siRNA) or S100A4‐targeting siRNA (siS100A4). Gene expression was normalized to GAPDH and presented relative to scrambled siRNA controls. Data are presented as mean ± SEM. Statistical significance was determined using a two‐tailed unpaired Student's *t*‐test: *p < 0.05.

## Data Availability

The data supporting the findings of this study are available from the corresponding author upon reasonable request.
