# Supplementary material for: S100A4 Orchestrates Fibroblast Fate to Drive Fibrotic Remodeling
Source: FASEB J. 2026 Jun 13;40(12):e72043. doi: 10.1096/fj.202600886R (PMC13264399; doi:10.1096/fj.202600886R)
Supplement: Supplementary file 1 — Figure S1: S100A4 attenuates hydrogen peroxide–induced apoptotic signaling in primary murine lung fibroblasts. Serum‐starved primary murine lung fibroblasts were pretreated with S100A4 (1 μg/mL) prior to exposure to hydrogen peroxide (H2O2, 200 μM). (A) Representative immunoblot showing cleaved caspase‐3 expression following H2O2‐induced oxidative stress ± S100A4 pretreatment. (B) Densitometric quantification of cleaved caspase‐3 normalized to GAPDH. Data are presented as mean ± SEM of three independent biological experiments (n = 3). Statistical significance was determined by One‐way ANOVA with Tukey's post hoc test. *p < 0.05 and **p < 0.01. Figure S2: S100A4 knockdown attenuates ERK phosphorylation in primary human IPF fibroblasts. Primary human IPF fibroblasts were transfected with scrambled siRNA (scr siRNA) or S100A4‐targeting siRNA (siS100A4). (A) Representative immunoblot analysis showing phospho‐ERK (p‐ERK) and total ERK expression. (B) Densitometric quantification of p‐ERK normalized to total ERK from (A). Bars represent mean ± SEM. Statistical significance was determined by One‐way ANOVA with Tukey's post hoc test. *p < 0.05. Figure S3: siRNA‐mediated S100A4 knockdown attenuates profibrotic gene expression in primary human IPF fibroblasts. RT‐qPCR analysis of (A) S100A4, (B) ACTA2, and (C) COL1A1 mRNA expression in IPF fibroblasts transfected with scrambled siRNA (scr siRNA) or S100A4‐targeting siRNA (siS100A4). Gene expression was normalized to GAPDH and presented relative to scrambled siRNA controls. Data are presented as mean ± SEM. Statistical significance was determined using a two‐tailed unpaired Student's t‐test: *p < 0.05. [file FSB2-40-e72043-s001.pdf]

Supplementary Figure S1

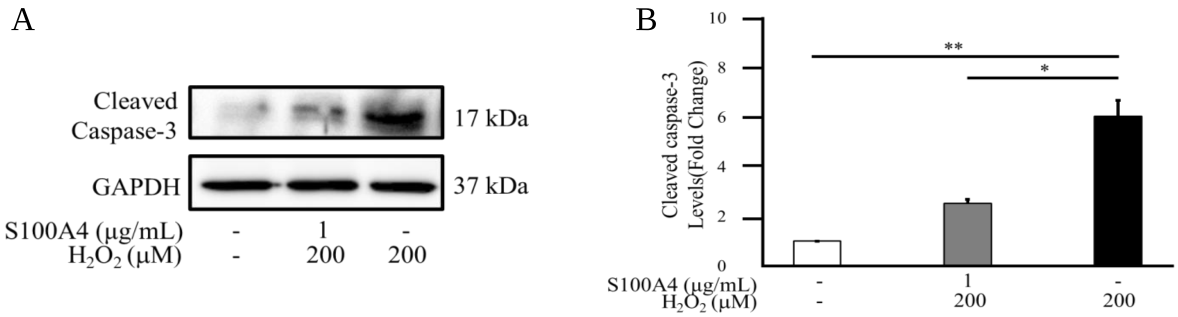

Supplementary Figure S1. S100A4 attenuates hydrogen peroxide–induced apoptotic signaling in murine lung fibroblasts. Serum-starved primary murine lung fibroblasts were pretreated with S100A4 (1 μg/mL) prior to exposure to hydrogen peroxide (H_2O_2, 200 μM).

(A) Representative immunoblot showing cleaved caspase-3 expression following H_2O_2-induced oxidative stress ± S100A4 pretreatment.

(B) Densitometric quantification of cleaved caspase-3 normalized to GAPDH. Data are presented as mean ± SEM of three independent biological experiments (n=3). Statistical significance was determined by one-way ANOVA with Tukey's post hoc test. *p<0.05 and **p<0.01

Supplementary Figure S2

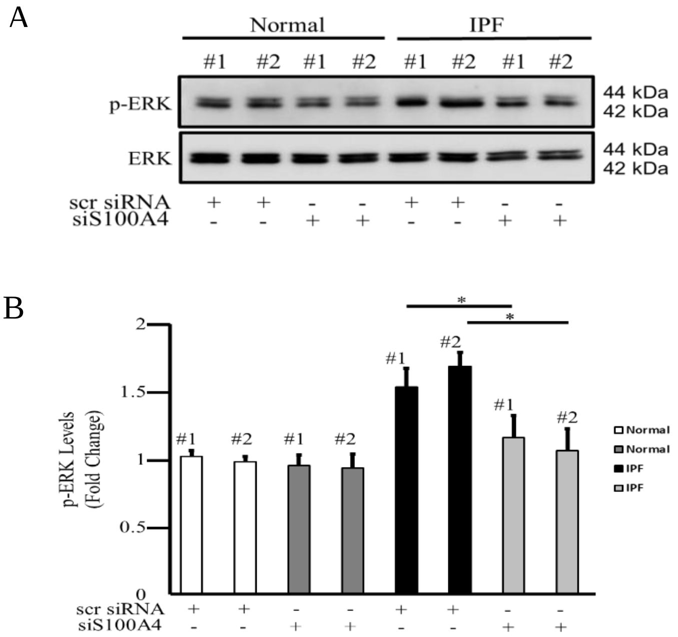

Supplementary Figure S2. S100A4 knockdown attenuates ERK phosphorylation in primary human IPF fibroblasts. Primary human IPF fibroblasts were transfected with scrambled siRNA (scr siRNA) or S100A4-targeting siRNA (siS100A4).

(A) Representative immunoblot analysis showing phospho-ERK (p-ERK) and total ERK expression.

(B) Densitometric quantification of p-ERK normalized to total ERK from (A). Bars represent mean ± SEM. Statistical significance was determined by one-way ANOVA with Tukey's post hoc test. *$p<0.05$.

Supplementary Figure S3

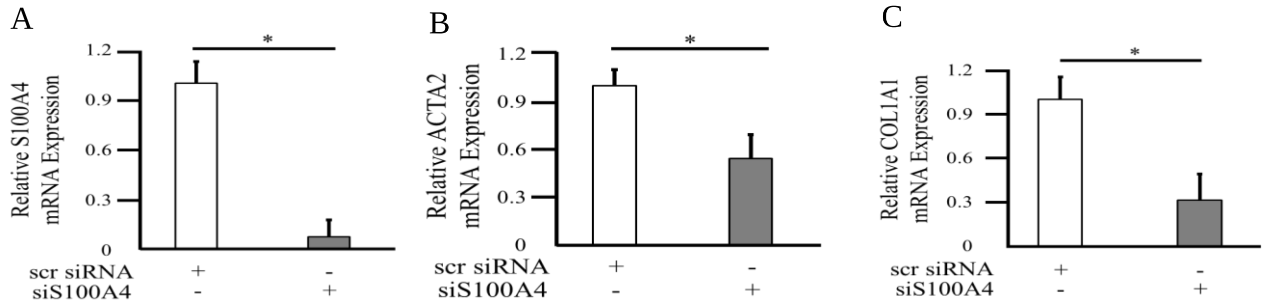

Supplementary Figure S3. siRNA-mediated S100A4 knockdown attenuates profibrotic gene expression in primary human IPF fibroblasts. RT-qPCR analysis of (**A**) S100A4, (**B**) ACTA2, and (**C**) COL1A1 mRNA expression in IPF fibroblasts transfected with scrambled siRNA (scr siRNA) or S100A4-targeting siRNA (siS100A4). Gene expression was normalized to GAPDH and presented relative to scrambled siRNA controls. Data are presented as mean ± SEM. Statistical significance was determined using a two-tailed unpaired Student's t-test: *$p<0.05$.
